# HERVOminer: a sequence similarity-based approach for recognizing endogenous retrovirus origin of the peptidome

**DOI:** 10.1038/s41698-026-01370-9

**Published:** 2026-03-18

**Authors:** Chia-Hsin Wu, Tsz Wun Fok, Kevin Chih-Yang Huang, Mong-Hsun Tsai, Liang-Chuan Lai, Tzu-Pin Lu, K. S. Clifford Chao, Eric Y. Chuang, Chien-Yueh Lee

**Affiliations:** 1 Bioinformatics and Biostatistics Core Lab, NTU Center of Genomics and Precision Medicine, Taipei, Taiwan; 2 Graduate Institute of Biomedical Electronics and Bioinformatics, National Taiwan University, Taipei, Taiwan; 3 Department of Biomedical Imaging and Radiological Science, China Medical University, Taichung, Taiwan; 4 Translation Research Core, China Medical University Hospital, China Medical University, Taichung, Taiwan; 5 Cancer Biology and Precision Therapeutics Center, China Medical University, Taichung, Taiwan; 6 Office of Research and Development, Asia University, Taichung, Taiwan; 7 Institute of Biotechnology, National Taiwan University, Taipei, Taiwan; 8 Graduate Institute of Physiology, College of Medicine, National Taiwan University, Taipei, Taiwan; 9 Institute of Health Data Analytics and Statistics, National Taiwan University, Taipei, Taiwan; 10 Proton Therapy and Science Center, China Medical University Hospital, China Medical University, Taichung, Taiwan; 11 Graduate Institute of Biomedical Science, China Medical University, Taichung, Taiwan; 12 Biomedical Technology and Device Research Laboratories, Industrial Technology Research Institute, Hsinchu, Taiwan; 13 Master Program in Artificial Intelligence Technology, Innovation Frontier Institute of Research for Science and Technology, National Taipei University of Technology, Taipei, Taiwan; 14 Department of Electrical Engineering, National Taipei University of Technology, Taipei, Taiwan

**Keywords:** Cancer, Computational biology and bioinformatics, Oncology

## Abstract

Determining tumor-specific antigens (TSAs) derived from human endogenous retrovirus (HERV) regions is crucial because HERVs are a promising source of shared TSAs. However, systematic tools for accurately tracing peptide origins are limited by the high homology of HERV sequences, the complexity of quantifying multi-mapping reads, and a prior emphasis primarily on the long terminal repeat. To this end, we developed a sequence similarity-based approach (HERVOminer) to identify the HERV origins of peptides from our predefined HERV open-reading frame (ORFs) databases. HERVOminer compared sequence similarity between ORFs and candidate peptides, quantified their expression to determine the most expressed HERV fragment, and visualized the results. In our investigation of 15 colorectal cancer cohorts, candidate TSAs with HERV origins were found to be abundantly expressed and widely shared. Using ELISpot, we confirmed the immunogenicity of the candidate TSA. Furthermore, applying HERVOminer to HERV-derived peptides from preclinical and clinical studies, with quantification in the colorectal cancer samples, revealed broader genomic origins, with higher expression in tumor tissues compared to normal tissues. HERVOminer is implemented as an interactive web interface and a command-line package. HERVOminer can accelerate the development of novel therapeutic interventions such as off-the-shelf cancer vaccines.

## Introduction

Tumor-specific antigens (TSAs) represent a class of antigens generated by tumor cells that result from genomic alterations, RNA dysregulation, and the integration of viral sequences^1^. Among these, neoantigens constitute a distinct subset of TSAs that most commonly arise from single-nucleotide variants (SNVs)^2^. These alterations give rise to novel peptide sequences that are processed and presented as peptide–major histocompatibility (pMHC) complexes on the surface of tumor cells. Ideally, T cells recognize these pMHC complexes via their T cell receptors, leading to their activation and differentiation into cytotoxic T lymphocytes (CTLs)^3^. Once activated, CTLs can eliminate tumor cells through mechanisms such as releasing perforin and granzymes, or engaging death receptors (e.g., FasL-Fas interaction), while also propagating antitumor responses via paracrine signaling^3,4^. Moreover, it is essential to distinguish neoantigens from tumor-associated antigens (TAAs), such as CD19, which are often normal proteins that are aberrantly overexpressed or restricted to specific lineages^5^. In contrast, neoantigens are exclusively expressed by tumor cells and are absent from normal tissues, providing an ideal foundation for effective personalized cancer treatment. However, the majority of neoantigens derived from SNVs often exhibit limited immunogenicity due to their high similarity to wild-type peptides, which subjects them to central tolerance mechanisms^6,7^. Furthermore, the patient-specific nature of these SNV-derived neoantigens is not favorable for the development of off-the-shelf cancer vaccines^2^. Thus, identifying alternative classes of TSAs that may provide better immunogenic properties is important, while maintaining high tumor specificity to minimize the risk of on-target off-tumor effects^8^.

Recently, beyond investigating the exonic regions of the genome, there has been a growing interest in aberrantly expressed TSAs (aeTSAs), mainly from the non-coding regions^9^. The aeTSAs result from epigenetic changes and unusual protein synthesis processes; they present promising targets for research and therapeutic intervention^9^. One example is human endogenous retroviruses (HERVs), which evolved from exogenous retroviruses by integrating into the germ line of their host and comprise up to 8% of the human genome^10^. As evolutionary changes accumulate, replication-competent HERVs gather in-frame stop codons and frame-shift mutations due to the replication processes of host DNA, eventually causing these elements to become inactive^11^. It has been suggested that in tumorigenesis, HERV regions are demethylated^10,12^; the consequent lack of ability to suppress or regulate gene expression causes these HERV regions to be aberrantly expressed and translated in tumor cells.

Because the aberrantly expressed HERVs are treated as foreign viral antigens by the human immune system and have restricted expression in normal cells, HERVs serve as a valuable potential source of shared TSAs^13^, which are commonly present in cancer patients. Systems like RepeatMasker^14^ have cataloged a collection of HERV regions. Nevertheless, the focus of these systems is mainly on repetitive genetic elements. They often only find the highly repetitive parts of the HERV, such as the long terminal repeats, and ignore other regions in the provirus sequence. To identify the aeTSAs derived from HERV sequences in cancers, our study focused on the entire proviruses and utilized reference sequences that met this comprehensive requirement. Moreover, several HERV-related studies have delved into the sequences and disease associations of HERVs, leading to the development of several databases, such as CancerHERVdb^15^ and ERVcancer^16^. Also, tools like hervQuant^17^ and ERVmap^18^ have emerged, specifically focusing on HERV expression quantification. However, most existing tools are not designed to address the high sequence homology among HERV regions, resulting in inaccurate quantification of reads that map to multiple genomic locations. To address these limitations specifically for HERV-based TSAs, we introduced the approach HERVOminer to recognize the HERV origin of the target peptide sequences. The approach utilized local databases constructed based on the HERV reference with sequence similarity search to determine whether a query peptide originates from a HERV region, quantifying its expression, and visualizing the result.

## Results

### Case study 1: identifying colorectal cancer TSAs in 15 Taiwanese patients

Colorectal cancer ranks as one of the top prevalent malignancies globally. It constitutes around 10% of all cancer incidence^19^ and stands as the second principal contributor to cancer-related mortality worldwide, indicating an urgent need for identifying better therapeutic targets. In this case, LC-MS/MS data and RNA-seq data from 15 colorectal cancer patients were utilized to illustrate the analysis workflow of HERVOminer for HERV-based TSAs identification. Due to HLA-A*11:01 being the dominant genotype in the Taiwanese population, associated MHC-presented peptides (MPPs) were extracted.

Following HERVOminer analysis, three MPPs aligning to HERV regions with RNA expression were selected for demonstration: P1 (VILPPQPPK), P2 (AVLLPQPPK), and P3 (GILLPQPPK). The number of HERV ORFs aligned with each MPP varied (P1: 69, P2: 1266, P3: 345). Through stringent filtering based on identity and alignment consistency of peptide length with considering the accumulated mutations of each sample, HERVOminer effectively eliminated most false-positive ORFs, retaining a consistent set of candidate HERV ORFs at the proteomic level (P1: 14, P2: 9, P3: 5). Corresponding genomic coordinates were also extracted for user exploration or quantitative analysis. Furthermore, HERVOminer quantified the expression levels of candidate HERV fragments using RNA-seq data.

**Fig. 1:**
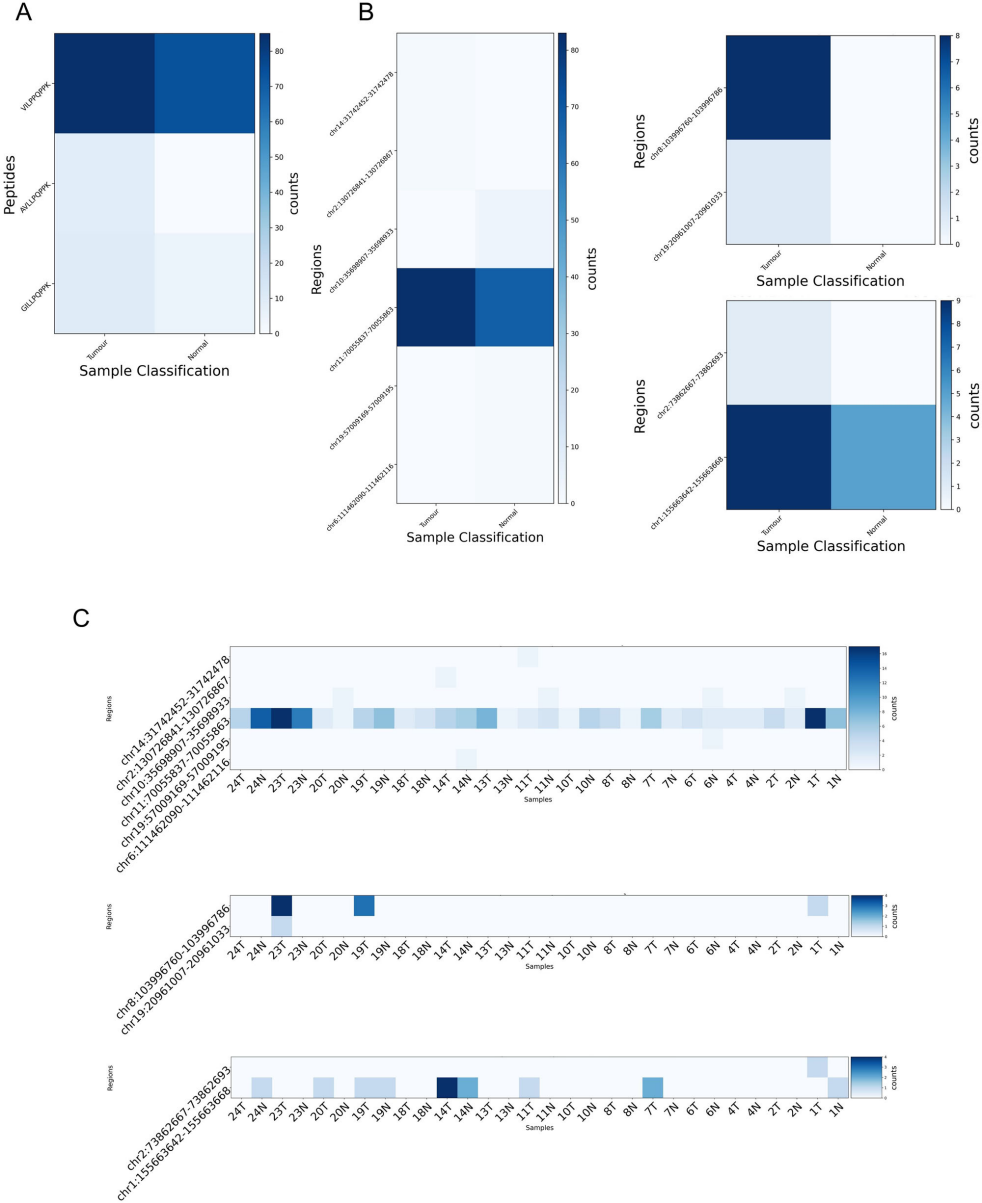
Heatmaps of expression quantification for each target peptide and potential HERV fragments identified from the 15 Taiwanese colorectal cancer patients. **A** Expression levels of target peptides between tumor and normal sample groups at the tissue level. **B** Expression levels of all HERV fragments encoding the target peptide between tumor and normal sample groups (left: VILPPQPPK, P1; upper right: AVLLPQPPK, P2; Lower right: GILLPQPPK, P3) at the tissue level. **C** Expression levels of all HERV fragments encoding the target peptide across all individual samples (top: VILPPQPPK, P1; middle: AVLLPQPPK, P2; bottom: GILLPQPPK, P3). The *x*-axis represents groups of tumor and normal samples in **A**, **B** and within each patient in **C**; the *y*-axis indicates target peptides in **A** and the DNA locations of all HERV fragments encoding the target peptide in **B**, **C**. The intensity of color indicates the number of reads (the darker color reflects more reads).

At the cohort level of tumor and normal samples, P1 exhibited the highest expression within HERV fragment (chr11:70,055,837-70,055,863; negative strand) (Table 1 and Fig. 1A, B), suggesting this locus as the most expressed origin for P1. This fragment aligned with 151 reads across both normal and tumor tissues, with 83 and 68 reads, respectively. Since this HERV fragment was also expressed in normal samples and with a substantial expression level, P1 might represent a TAA. Conversely, P2 displayed elevated expression within a HERV fragment (chr8:103,996,760-103,996,786; positive strand) predominantly in tumor samples at the cohort level (Table 1 and Fig. 1B), possibly indicating a TSA. At the individual level, three patients showed expression of P2, suggesting a potential shared TSA. One additional HERV fragment was expressed in patient 23 (Fig. 1C middle), and was only present in the tumor sample. In addition to transcriptomic evidence, users can also obtain the DNA sequences of both fragments from HERVOminer for further verification. In the analysis of P3, at the normal and tumor tissue level of expression, the HERV fragment (chr1:155,663,642-155,663,668; negative strand) emerged as the most expressed origin (Table 1 and Fig. 1B). However, similar to the analysis of P1, this HERV fragment might generally represent a TAA due to its expression in normal tissues. At the individual level, patients 7, 11, and 20 potentially harbored a TSA (Fig. 1C bottom), necessitating further validation and exploration of shared TSA potentiality. Notably, patient 1 exhibited expression in another HERV fragment, suggesting an additional potential origin for subsequent examination.

**Table. 1:** The most expressed HERV fragment per query peptide at the cohort level (colorectal cancer patients)

Peptide	TSA	HERV region	Tumor reads	Total reads	Strand	Validation-ready sequence*
P1	VILPPQPPK	chr11:70055837-70055863	83	151	–	TTTTTTTAGAGATGAGGTTTCCCTATGTTGGCGAGGCTGGCCTCAAACTCCTGGGTTCAAGTAATCCTCCCACCTCAGCCTCCCAAAGTGCAGGGATTACAGATGAGAGCCACTGCACCTGGCCTAGCGCCCAGTTTTAATTGAGG
P2	AVLLPQPPK	chr8:103996760-103996786	8	8	+	CAAGCTGGAGTGCAGTGGCACAATCTCGGGTCACTGCAACCTCCGCCTCCCAGATTCAAGCAGTTCTCCTGCCTCAGCCTCCCAAATAGCTGGGATTACAGGCACCTGCCACCATGTCTGGCTAAATTTTTGTATTTTTTTAGTAG
P3	GILLPQPPK	chr1:155663642-155663668	9	14	–	ACAGGCTGGAGTGCAATGGCGCAATCTCAGCTCACTGCAACCTCCGCCCCCCAAGTTCAAGGGATTCTCCTGCCTCAGCCTCCCAAGTAGCTGCGATTACAGGCATGTGCCACCACACCCTGCTAATTTTGTATTTTTAGTAGAGA

^*^ Underline represents the corresponding DNA sequences of candidate peptides.

**Fig. 2:**
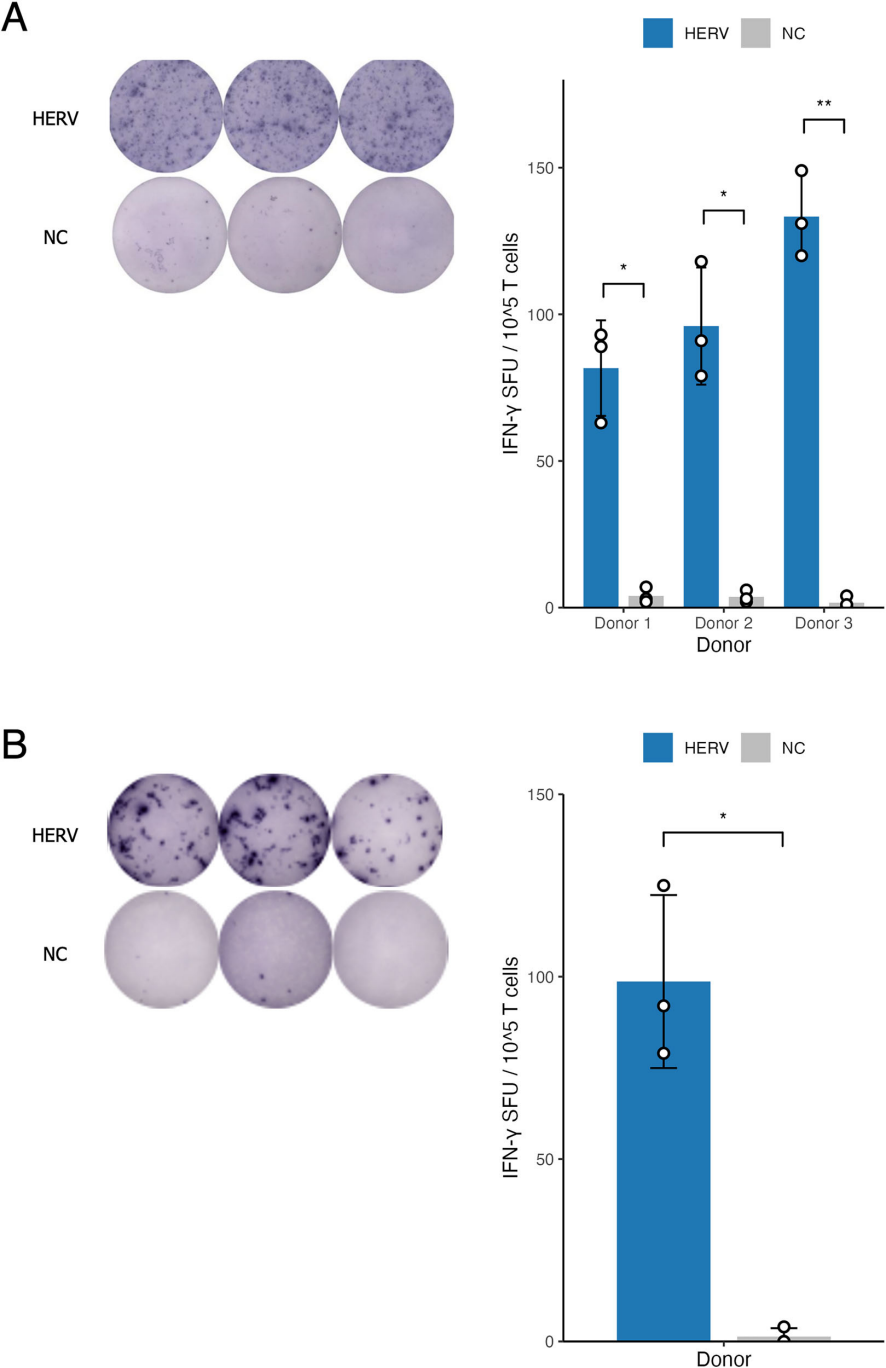
Immunogenicity evaluation of the HERV-based TSA identified by HERVOminer. The immunogenicity was evaluated by ELISpot assay of the TSA-specific IFNγ from HLA-A*11:01 human normal peripheral blood mononuclear cells (PBMCs). **A** P1 (VILPPQPPK) was assessed using PBMCs of three healthy donors. **B** P3 (GILLPQPPK) was assessed using PBMC of one healthy donor. The bar charts represent the number of IFNγ spots, demonstrating a significantly elevated response of CD8+ T cells to aeTSA compared to the negative control (NC) in each biological replicate. ^*^p < 0.05. ^**^p < 0.01. Unpaired *t* test.

To validate the immunogenicity of identified HERV antigens, an ELISpot assay was performed using peripheral blood mononuclear cells (PBMCs) from three healthy individuals with the HLA-A*11:01 genotype (Fig. 2). The results showed an increased response of HERV antigen-specific T cells within the PBMC population stimulated with P1 and P3, indicating that HERVOminer can assist users in identifying HERV antigens with potential immunogenicity. P2 was excluded from the ELISpot assay due to the low read counts in the sequencing data.

**Fig. 3:**
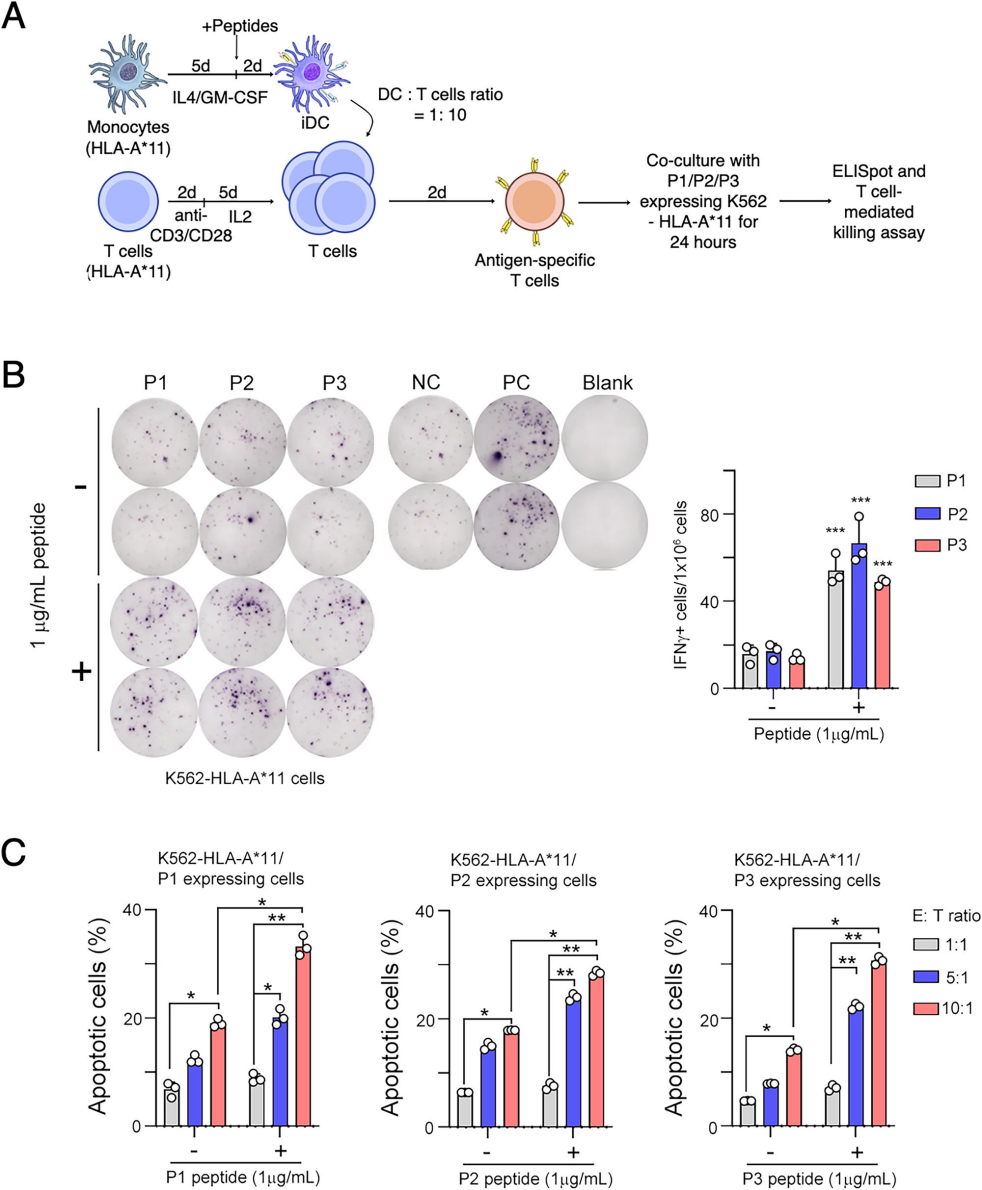
Validation of HERV antigen-based T cell-mediated killing ability ex vivo. **A** Immunogenicity was evaluated by ELISpot assay using a co-culture of generated antigen-specific T cells from HLA-A*11 healthy donor and K562-HLA-A*11 cells. **B** The frequencies of IFNγ + T cells were quantified and compared between peptide-pulsed (+) and unpulsed (-) groups. **C** T cell-mediated cytotoxic ability was assessed by measuring the percentage of apoptotic cells in P1/P2/P3 minigene expressing K562-HLA-A*11 cells. Apoptosis levels were quantified and compared between peptide-pulsed and unpulsed groups at different effector-to-target (E:T) ratios for each peptide (P1, P2, and P3). ^*^p < 0.05. ^**^p < 0.01. ^***^p < 0.001.

Furthermore, we generated antigen-specific T cells from HLA-A*11 healthy donor via ex vivo stimulation protocol (Fig. 3A), and evaluated the IFN-γ+ T cell via an ELISpot assay. Additionally, we individually generated P1/P2/P3 minigene expressing K562-HLA-A*11 cells, and then co-cultured peptide-pulsed P1/P2/P3 minigene expressing K562-HLA-A*11 cells with antigen-specific T cells for 24 h for ELISpot and T cell-mediated killing assay. All three peptides (P1, P2, and P3) showed a statistically significant increase in the frequency of IFN-γ+ secreting cells compared with control groups (Fig. 3B). Moreover, we measured the T cell-mediated killing ability in peptide-pulsed K562-HLA-A*11 cells with different effector-to-target (E:T) ratios. We observed a significant increase in apoptotic cell percentage at the 10:1 ratio compared to the control group. Furthermore, the apoptotic peptide-pulsed K562-HLA-A*11 cells exhibited a significant increase as the effector-to-target ratio from 1:1 to 10:1 (Fig. 3C).

### Case study 2: evaluation of clinical trials-validated peptides in 15 Taiwanese colorectal cancer patients

To validate the comprehensiveness and functionality of HERVOminer, we applied four HERV peptides reported in three external studies to our cohort of 15 Taiwanese patients with colorectal cancer. The sequences of the four peptides were: PO1 (ATFLGSLTWK)^20^, PO2 (ATFLGSLTGK)^21^, PO3 (KLIAGLIFLK)^21^, and PO4 (NEAIEQVRAICLRAW)^22^. These studies focused on kidney cancer, clear cell renal cell carcinoma, and ovarian cancer, respectively.

First, to evaluate the comprehensiveness of HERVOminer, we compared the genomic coordinates of the candidate HERV ORFs identified by the tool against the “ground truth” regions specified in the literature. Following stringent filtering based on peptide identity and length, while accounting for the accumulated mutations within each sample, all four peptides were successfully mapped to and expressed in multiple HERV ORFs. Crucially, this mapping included ORFs located within the exact ground truth regions cited in the literature (PO1: chr6:89372306-89372335, PO2: chr20:15966388-15966417, PO3: chr20:15966388-15966417, PO4: chr8:7362476-7362520).

**Fig. 4:**
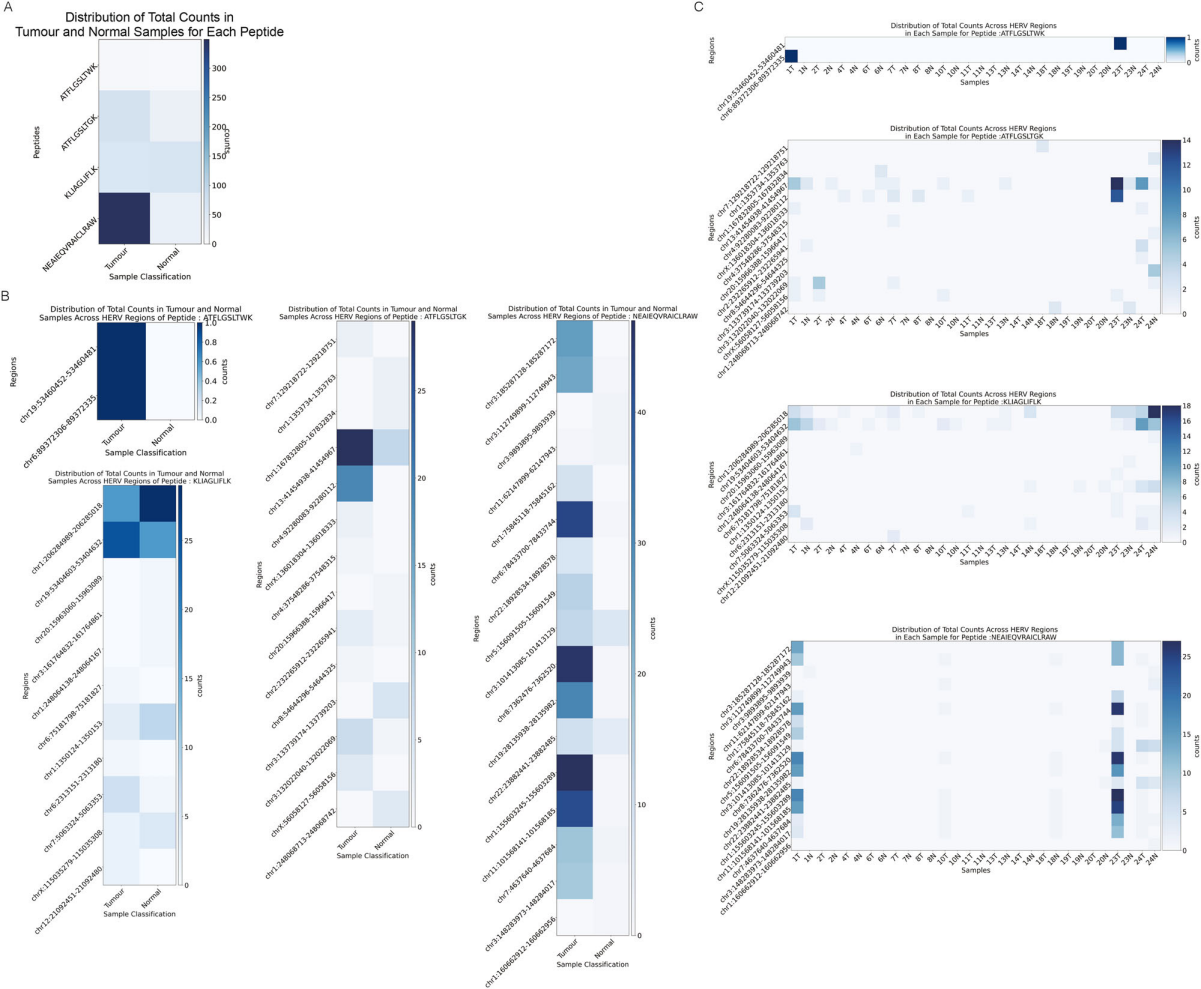
Heatmaps of expression quantification for clinical trials-validated peptides and potential HERV fragments using the colorectal cancer cohort. **A** Expression levels of target peptides between tumor and normal sample groups at the tissue level. **B** Expression levels of all HERV fragments encoding the target peptide between tumor and normal sample groups (upper left: ATFLGSLTWK, PO1; middle: ATFLGSLTGK, PO2; lower left: KLIAGLIFLK, PO3; right: NEAIEQVRAICLRAW, PO4) at the tissue level. **C** Expression levels of all HERV fragments encoding the target peptide across all individual samples (top: ATFLGSLTWK, PO1; second: ATFLGSLTGK, PO2; third: KLIAGLIFLK, PO3; bottom: NEAIEQVRAICLRAW, PO4). The *x*-axis represents groups of tumor and normal samples in **A**, **B** and within each patient in **C**; the *y*-axis indicates target peptides in **A** and the DNA locations of all HERV fragments encoding the target peptide in **B**, **C**. The intensity of color indicates the number of reads (the darker color reflects more reads).

Furthermore, to access clinical applicability and expression profiling, we quantified the expression of the mapped ORFs within our cohort. All four peptides were found to be expressed. At the cohort level, PO4 exhibited the highest overall expression in tumor samples (Table 2). Additionally, PO1, PO2, and PO4 showed higher total tumor reads compared to normal reads (Table 2 and Fig. 4A).

**Table. 2:** The most expressed HERV fragment of clinical trials-validated peptides at the cohort level (colorectal cancer patients)

Peptide	TSA	HERV regions	Tumor reads	Total reads	Strand	Validation-ready sequence*
PO1	ATFLGSLTWK	chr19:53460452-53460481	1	1	+	AATAAAGCCACTTCCTTCTTTAATCCGTTGTCTGAGAGGTACTGTCTGCGGCTCGTCATGCTACATTTCTTGGTTCCCTGACTTGGAAGCGAGGTAATTAACAGAAGGTCGAGGCAACCCCTTAGGTGACTTAGGCCTGCCCTTTGGAG
PO2	ATFLGSLTGK	chr13:41454938-41454967	29	37	–	GAATAAAGCTACTTCCTTTCTCAACCTGGTGTCTGAGGGGTTTTGTCCACAGCTTGTCCTGCTACATTTCTTGGTTCTCTGACTGGGAAGCGAGGTGATTAGCAGACAGTCAAGGCAGCCCCTTAGGTGGCTCAGGCCTGCCCTGTGGA
PO3	KLIAGLIFLK	chr1:206284989-206285018	17	46	–	CAAGCCCTGCTCCAGTCACACCCGGAAGCTGACTGGTCCACGCACAGCTGAAGCATGAGGAAACTCATCGCGGGACTAATTTTCCTTAAAATTTAGACTTGCACAGTAAGGACTTCAACTGACCTTCCTCAGACTGAGAACTGTTTCCA
PO4	NEAIEQVRAICLRAW	chr1:155603245-155603289	47	48	–	GATCAACTATTAGGAATAGGTCAAAATTGGAGTACTATTAGTCAACAAGCATTAATGCAAAATGAGGCCATTGAGCAAGTTAGAGCTATCTGCCTTAGAGCCTGGGAAAAAATCCAAGACCCAGGAAGTACCTGCCCCTCATTTAATACAGTAAGACAAGGTTC

^*^ Underline represents the corresponding DNA sequences of candidate peptides.

Individually, PO1 mapped to two HERV ORFs and generally displayed low expression across all samples (Table 2). In contrast, PO2 mapped to 14 HERV ORFs, with the highest expressing ORF localized at chr13:41454938-41454967 (Table 2). PO3 mapped to 13 ORFs, with the region of the highest expression located at chr1:206284989-206285018 (Table 2). Finally, PO4 mapped to 17 ORFs, and the coordinates corresponding to the most expressed ORF were chr1:155603245-155603289 (Table 2).

### HERVOminer usage

**Fig. 5:**
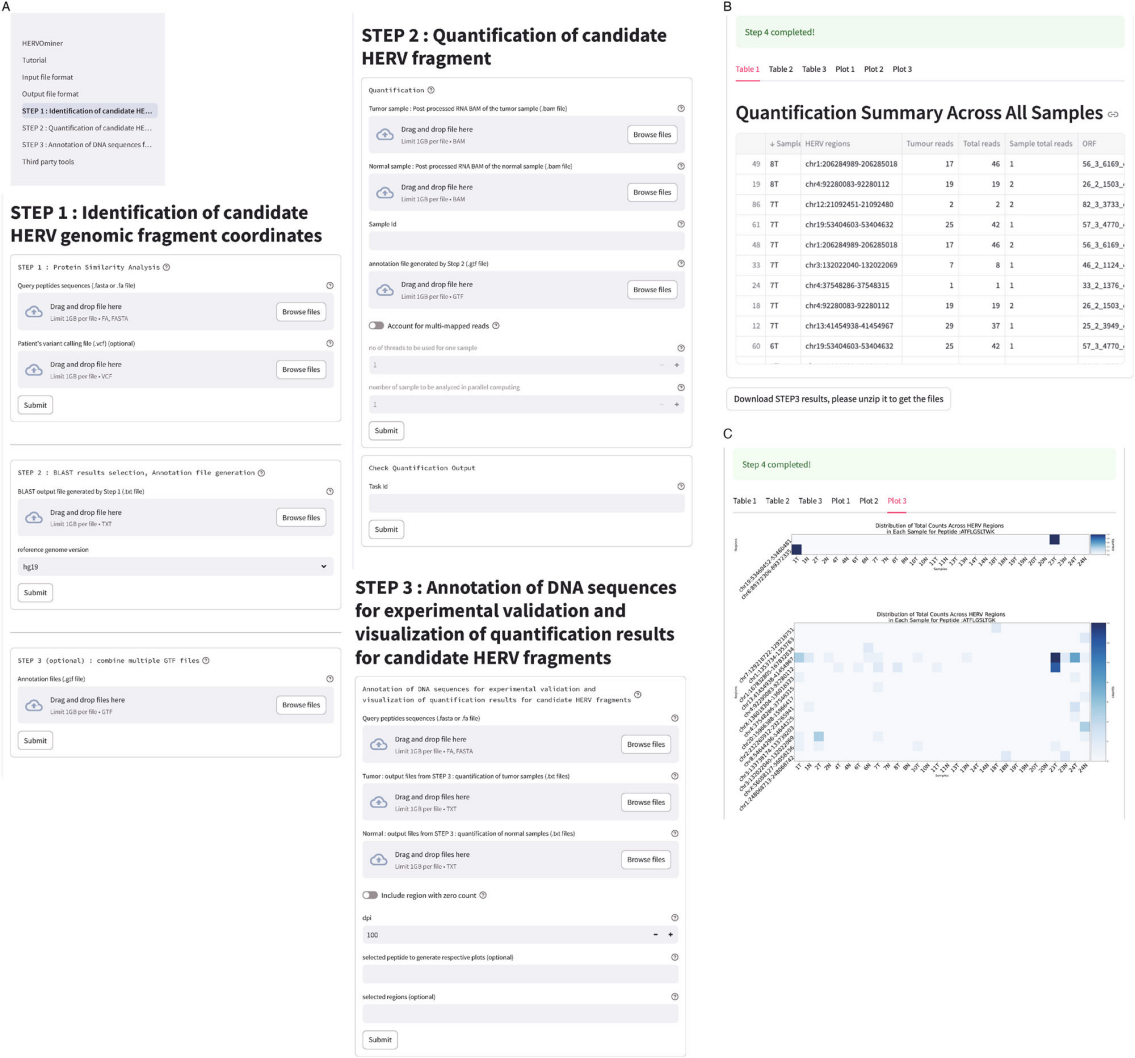
The screenshots of HERVOminer web interface. **A** Screenshots of the analysis panel. This panel describes the three main steps of the HERVOminer pipeline and displays links to input and output file formats and the tutorial. HERVOminer takes the inputs of peptides and RNA-seq data for each sample separately in the first two steps. In the final step, users can submit an arbitrary number of output results from step 2, and HERVOminer subsequently combines these for annotation and visualization. Users can follow the tutorial step by step to test whole functionalities using provided example data and easily interpret results. **B**, **C** Screenshots of the output tables and visualization. Query results are displayed as easy-to-use data tables and heatmaps, facilitating clear interpretation.

HERVOminer is designed to identify whether a queried peptide originates from HERV regions and aids users in determining the most reliable candidate origin, annotating the DNA sequence context for further experimental validation. In addition to the command-line interface, an interactive interface was developed using Streamlit to provide a user-friendly and accessible analysis platform for users with limited programming experience or computational resources. This interface guides users step-by-step through the analysis process (Fig. 5A), which starts the analysis by simply uploading a FASTQ file containing peptide sequences of interest and the post-processed RNA Binary Alignment/Map (BAM) files of the samples.

In Step 1, HERVOminer aligns the queried peptides to two local HERV databases. This step provides a Gene Transfer Format (GTF) file, which records matched HERV fragments with detailed genomic coordinates. In Step 2, users can check whether these candidate fragments are expressed. HERVOminer will assign a task ID to track the progress of this step, performing computations in parallel and ultimately providing quantification results at the individual or cohort levels. Step 3 offers individual and cohort-level visualization of expression levels for all candidate HERV fragments, specifically designed to complement Step 2. Users can decide whether to include HERV fragments with zero counts. Ultimately, this step outputs relevant heatmaps (Fig. 5B, C), allowing users to intuitively identify the most reliable HERV origins for each queried peptide. HERVOminer also provides the DNA sequence context of these fragments, facilitating subsequent experimental validation. Comprehensive instructions, including input and output descriptions and examples, are available on the web interface.

## Discussion

In recent years, HERV-related studies have advanced significantly, leading to the development of numerous dedicated databases and tools. Comprehensive resources such as CancerHERVdb^15^ and ERVcancer^16^ serve as established web-based databases, enabling convenient querying on pre-existing HERV expression profiles and their associations with various cancers. Furthermore, prevalent computational methodologies such as hervQuant^17^ and ERVmap^18^ were developed to facilitate the mapping of RNA sequences to HERV loci and to quantify locus-specific HERVs expression from RNA-seq data. However, these methods lacked the functionalities to query peptides originating from HERV regions or to compare the paired RNA-seq data. Our approach, HERVOminer, leverages existing databases of HERV locations and also possesses the critical functionality of quantifying HERV fragments. Moreover, HERVOminer is distinctive due to its unique capability of integrating paired transcriptome data and immunopeptidome data to identify HERV-based antigens. HERVOminer incorporates a comprehensive reference of 3173 HERV sequences and emphasizes alignment accuracy. Notably, HERVOminer aligns peptides to potential ORF fragments through its two local databases and subsequently maps the ORFs to DNA coordinates. This approach can avoid the issue of aligning peptides directly to DNA sequences, which may not correspond to ORF regions.

In the colorectal cancer case study, HERVOminer effectively screened LC-MS/MS data for HERV-related antigens and quantified the expression of candidate fragments using RNA-seq data to identify reliable HERV origins. The immunogenicity of the candidate was validated through an ELISpot assay with PBMCs from healthy individuals, demonstrating the robustness of HERVOminer in identifying immunogenic antigens. In addition, immunogenicity prediction tools, such as DeepHLApan^23^, are recommended for further selection. Furthermore, cell-based co-culture ELISpot and T cell-mediated killing assays demonstrated that the DNA sequences provided by HERVOminer for each candidate peptide were successfully processed and presented as antigens for T cell recognition. The generated antigen-specific T cells demonstrated both cytokine secretion and cytotoxic ability to eliminate tumor cells. The significant increase was observed specifically in the peptide-pulsed group; the possibility of non-specific activation was excluded. These results validated the functional utility of HERVOminer in providing accurate HERV genomic origins, thereby facilitating the systematic experimental validation of immunogenic HERV-derived antigens.

In case study 2, we validated the comprehensiveness, clinical applicability, and expression profiling capabilities of HERVOminer, while also assessing the tool’s ability to discover new genomic insights. We successfully mapped clinical trials-validated HERV peptides to the HERVOminer local database, which showed that the tool is both comprehensive and widely applicable. Subsequent quantification of these mapped regions within our colorectal cancer patient cohort confirmed that these peptides were expressed, with most exhibiting higher expression in tumor tissue than in normal tissue. Notably, we observed that the majority of these expressed peptides originated from genomic coordinates distinct from the regions reported in the original studies. This suggests a rich and diverse landscape of genomic loci capable of generating these HERV peptide sequences within the colorectal cancer patients, thereby underscoring their potential as immunotherapeutic targets for this disease. Moreover, given that the demethylation and overexpression of HERVs could potentially initiate several diseases, including rheumatoid arthritis, systemic lupus erythematosus, and multiple sclerosis, HERVOminer can also be applied to research beyond cancer. HERVOminer offers a user-friendly web interface via the Streamlit framework available at https://hervominer.cgm.ntu.edu.tw, enabling interactive analysis and visualization, while also providing a command-line-based package for local use.

HERVOminer had three limitations. While assessing an individual’s accumulation of mutations during the identification of candidate HERV genomic fragment coordinates, only SNVs were taken into account; longer mutations, such as insertions and deletions, were not considered. Since genome coordinates for the hg38 and T2T assemblies were lifted over from the hg19 assemblies, novel HERV regions present only in hg38 or T2T were not included. Moreover, several studies have shown that HERV fragments can be widely expressed in normal tissue^24,25^. Therefore, we did not specify a criterion based on the ratio of tumor to normal reads for reporting the candidate HERV fragment.

In summary, HERVOminer reduces the difficulty of identifying effective TSAs in multi-omics data and simplifies the selection of viable targets from MS-defined immunopeptidomes. We believe that HERVOminer will facilitate the development of HERV-based TSA immunotherapies, reducing on-target off-tumor effects and accelerating the development of cancer vaccines for diverse populations.

## Methods

**Fig. 6:**
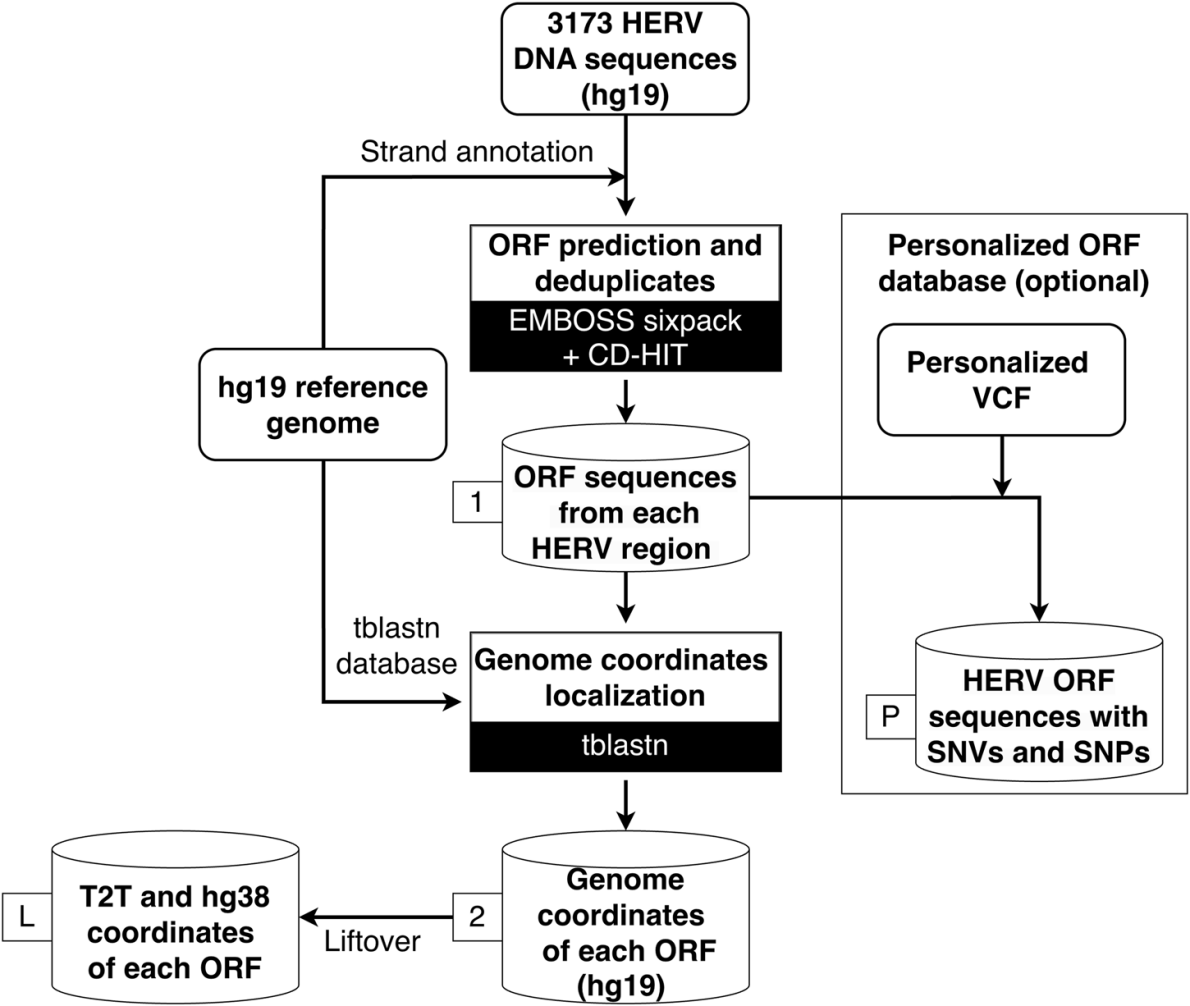
An overview of HERVOminer database construction. The detailed database construction workflow can be divided into two parts. The 3173 human endogenous retrovirus (HERV) DNA sequences were initially annotated by strand information. For the HERV open reading frame (ORF) database, ORFs were predicted by HERV DNA sequences using the EMBOSS sixpack package, and CD-HIT was subsequently used to reduce duplicate ORFs. For the genome coordinate database of HERV ORFs, the tblastn tool was used to align the non-duplicate ORFs against their corresponding DNA sequences to identify precise positions in the genome. The coordinates of the HERV ORFs were lifted over from the hg19 assembly to the hg38 and T2T assemblies. Users can build a personalized ORF database by uploading Variant Calling Format (VCF) files for each sample. (1: first database, 2: second database, P: personalized first database, L: liftovered database).

HERVOminer is a sequence similarity-based approach that is primarily designed to analyze MPPs. It traces all potential HERV genomic fragments from each target peptide, followed by integrating RNA sequencing (RNA-seq) data from samples to quantify the expression for each annotated fragment. Finally, HERVOminer can assist users in determining the HERV origins for the target peptides and provide experimental validation information for further interpretation of data. An interactive web interface designed with Streamlit and a command-line interface integrating the HERVOminer approach were released. The specifications of the pre-established reference database (Fig. 6), main analysis functionalities and steps, and example datasets are elaborated below.

### Constructing the local databases

This study selected the most comprehensive HERV reference (hg19)^26^, comprising a total of 3173 regions identified by screening the hg19 genome sequences with RetroTector^27^, to construct two necessary databases for the HERVOminer analytical process. Each HERV sequence was initially aligned with the corresponding hg19 genome sequences to annotate the forward or reverse strand information, ensuring the accuracy of subsequent alignments and streamlining the process (Fig. 6).

The construction of two HERVOminer local databases, an open reading frame (ORF) database and a genome coordinate information database, was achieved as follows: (1) To precisely trace HERV fragments encoding the target peptides during analysis, the EMBOSS sixpack was employed to predict the potential ORFs of each HERV region in the reference with three forward and three reverse translations^28^. For each region, CD-HIT^29^ is utilized to reduce duplicate ORFs, thereby establishing a database for protein BLAST^30,31^. (2) We implemented tblastn^30,31^ to align the ORFs to their corresponding DNA sequences for localizing coordinates in the reference genome. (3) The genomic coordinates were lifted over to the hg38 and T2T assemblies, and the results were organized and stored in a dictionary-format database. Once we matched the ORFs, we could determine the location, thereby aid in providing subsequent experimental validation with DNA sequences.

### Loading the input data

HERVOminer can trace common HERV region-translated peptides from multiple patient samples based on target query peptides. Users only need to submit two inputs for HERVOminer: (1) a post-processed RNA BAM file for the samples and (2) a FASTA file containing the peptide sequences of interest.

### Main analysis functionalities and steps

**Fig. 7:**
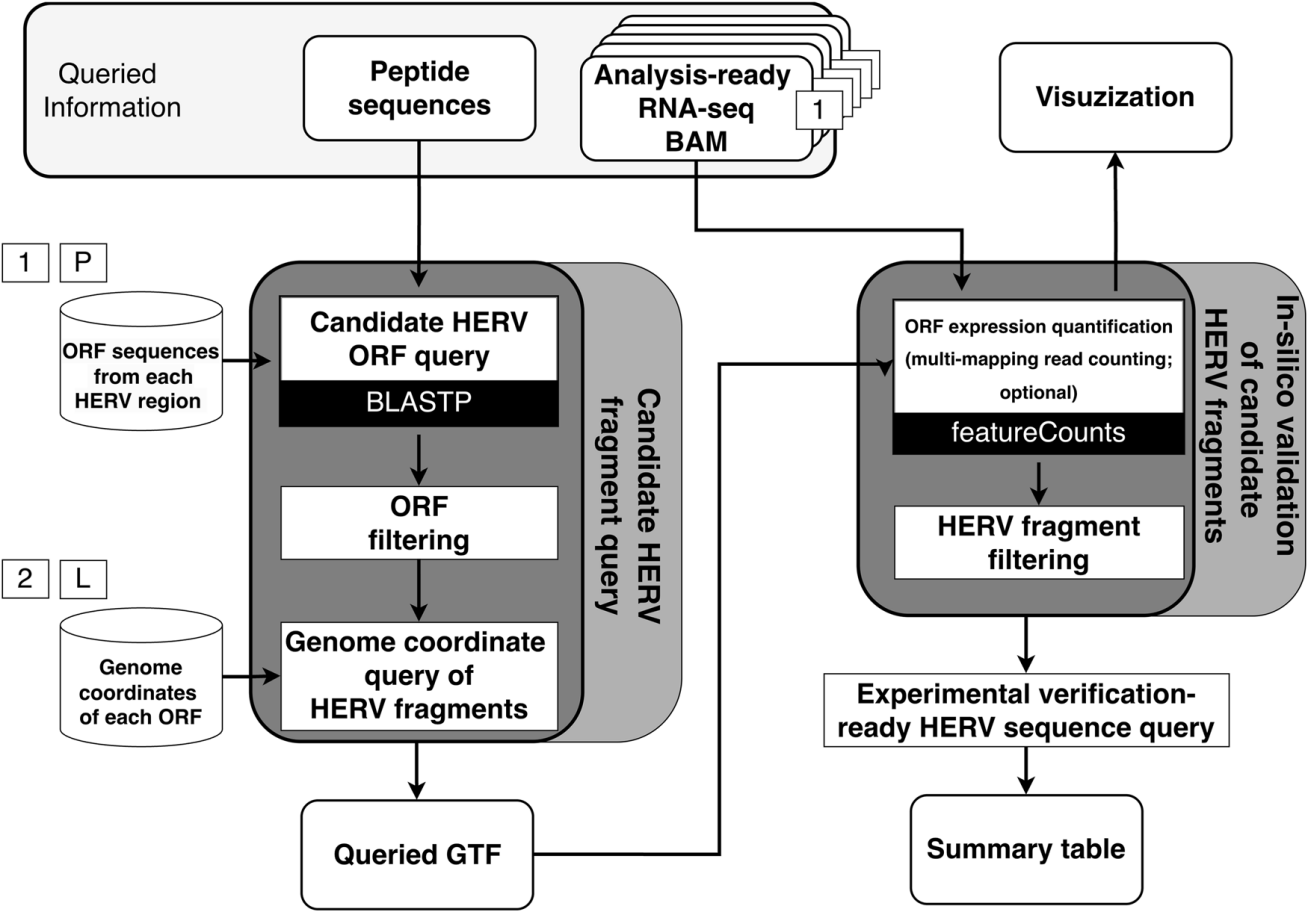
Schematic of the overall design of HERVOminer. Queried peptides are initially aligned to the human endogenous retrovirus (HERV) open reading frame (ORF) database using BLASTP, followed by matching corresponding genome coordinates. This formats the output into a Gene Transfer Format (GTF) file. The featureCounts tool is subsequently used to quantify candidate HERV fragments based on RNA sequencing (RNA-seq) data and the GTF file, and HERVOminer then provides visualization of the quantified results. Finally, full length DNA sequences of candidate HERV ORFs are annotated for experimental validation, followed by formatting into summary tables. BAM: binary Alignment/Map. (1: first database, 2: second database, P: personalized first database, L: liftovered database).

HERVOminer is capable of providing the GTF files containing all potential HERV genomic fragments encoding the target peptides (Fig. 7). Furthermore, a data table is generated to summarize read counts for each candidate HERV fragment from every sample, accompanied by annotation of the corresponding DNA sequence contexts to facilitate experimental validation. HERVOminer can further identify the most likely HERV fragment candidates for each target peptide at the cohort or individual level, providing two summarization tables and visualization plots. The delineated analytical functions are outlined below.

Initially, HERVOminer is utilized to identify candidate HERV genomic fragment coordinates. Target peptides are aligned to the local ORF database of HERV regions using Protein BLAST to calculate regions of similarity. HERVOminer also allows users to build an ORF database for each sample while considering the accumulation of mutations. By providing a Variant Call Format file, HERVOminer tracks mutations that overlap with the HERV region and adjust the affected ORF sequences within the local ORF database. Default values of the following protein BLAST parameters are changed: “-word_size 3 -gapopen 9 -gapextend 1 -matrix PAM30 -threshold 16 -comp_based_stats 0 -window_size 15 -num_threads: 48 -evalue 0.05 -outfmt ‘6 qacc sacc pident qseq length mismatch gapopen qlen qstart qend slen sstart send evalue bitscore’.” Selection is based on identity and alignment length, obtaining the positions of target peptides on all candidate HERV ORFs. HERV fragments that are not 100% matched or have different lengths with the query peptides are filtered. Finally, the coordinates of candidate HERV genomic fragments for the target peptides are determined by querying the genomic coordinates database of HERV ORFs. The identified fragments are organized into a temporary file based on the Protein BLAST and tblastn output files with three new fields, including the strand of the search results, the position of the whole ORF sequence, and the position of the aligned search results. The hg19, hg38 and T2T genome assemblies were available as options for users. The output annotation file is in GTF format and contains the detailed annotation of genetic features of the corresponding HERV region of the BLAST results.

In the subsequent stage, candidate HERV fragments are quantified. This step integrates RNA-seq BAM files from the samples and genomic positions recorded in the GTF file to quantify expression levels using featureCounts^32^. Concurrently, it calculates the read counts for each sample under different candidate HERV fragments. Users could also choose whether to account for multi-mapped reads in the samples by fractionally counting each alignment. After obtaining individual-level data, grouping by tumor and normal samples yields cohort-level quantification results. Subsequently, based on read counts under HERV fragments, the most expressed fragments can define the most expressed HERV origins at different levels based on tumor and normal samples. To address the low efficiency of the single-file quantification process employed by featureCounts, parallel processing capabilities have been integrated into HERVOminer. This enables HERVOminer to manage multiple quantification tasks simultaneously and improve throughput.

For each candidate HERV fragment, full-length DNA sequences of candidate HERV ORFs are annotated for experimental validation (Fig. 7). Users can further opt to exclude results with zero counts. Three data tables generated from the quantification and annotation results include (1) a summary table for target peptides across all samples, which contains read counts for each candidate HERV fragment and validation-ready DNA sequences; (2) the most expressed HERV fragment per query peptide at the tissue level, which identifies fragments with the highest total counts for each query peptide, counted by summing read counts from all tumor and normal samples, respectively; and (3) the most expressed HERV fragment for each query peptide by sample. This table shows the HERV region with the highest count in the individual sample level for each peptide and displays ‘no read’ for samples where the specific query peptide has a zero count.

Finally, quantification results for candidate HERV fragments are visualized. Individual and cohort-level visualization of expression levels for all candidate HERV fragments of each target peptide or the most expressed HERV fragments for each target peptide, is achievable. HERVOminer provides three kinds of heatmaps: (1) expression levels of target peptides between tumor and normal sample groups at the tissue level, counted by summing read counts from all candidate fragments and grouped by tumor and normal samples; (2) expression levels of all HERV fragments encoding the target peptide between tumor and normal sample groups at the tissue level, counted by corresponding HERV fragments and grouped by tumor and normal samples to show the count differences across these two sample types for each peptide; and (3) expression levels of all HERV fragments encoding the target peptide across all individual samples to show the count differences across patient samples for each peptide.

### Example datasets

This study includes an example dataset to demonstrate the accessibility and robustness of HERVOminer in identifying HERV origins. The dataset primarily comprised 15 participants diagnosed with colorectal cancer recruited from China Medical University, Taiwan. The research protocol underwent review and approval by the Institutional Review Board (IRB) of the hospital [Protocol number: CMUH110-REC3-014], and performed in accordance with the Declaration of Helsinki and its later amendments. All eligible participants provided informed consent prior to any study procedures or sample processing. Each patient had RNA-seq data for both tumor and matched normal tissues. First, quality control of RNA-seq reads was performed by FastQC (http://www.bioinformatics.babraham.ac.uk/projects/fastqc/), followed by Trimmomatic^33^ for trimming the low-quality and adapter sequences. Subsequently, the RNA-seq reads were mapped with STAR v2.7.3a^34^ to the hg19 reference transcriptome compiled with the annotation of 3173 HERV sequences. Peptide data were obtained from liquid chromatography-tandem mass spectrometry (LC-MS/MS), primarily capturing MPPs.

### ELISpot assay

IFNγ ELISpot assay was employed to validate that the HERVOminer-identified HLA-A*11:01 HERV antigen was immunogenic. Human normal PBMCs from health donors were plated and stimulated with a single 25-mer TSA peptide at 1 μg/mL concentration overnight. Negative and positive controls were implemented using peptide diluents dimethyl sulfoxide (Sigma–Aldrich) and concanavalin A (ConA; Sigma–Aldrich), respectively. Subsequently, a series of incubation steps was carried out for plates, including biotinylated anti-IFNγ, streptavidin-alkaline phosphatase conjugate, and 5-bromo-4-chloro-3-indolyl-phosphate/nitro blue tetrazolium 1-Step solution for development (Thermo Fisher Scientific). The ELISpot assay results were evaluated with an automated video analysis system and a plate reader, quantifying IFNγ spot-forming cells per million human PBMCs. Criteria for positive responses included: (i) detection of IFNγ production in ConA-stimulated wells, (ii) presence of at least 30 spots per million human PBMCs, and (iii) positive spots observed at least three times higher than the number of control spots.

### Recombinant lentivirus carrying colorectal cancer hotspot minigenes and HLA-A*11:01/hB2M

K562 cells in the logarithmic growth phase were infected with pLVX-HLA-A*11/hB2M-hygromycin. Hygromycin (Invitrogen, San Diego, CA, USA) was used for positive selection of transfected cells. Human shared TSA minigenes were constructed as described previously^35^. Minigenes were individually constructed for the HERV-derived peptides (P1, P2, and P3). These genes encoded the mutated amino acid and surrounding upstream and downstream native amino acids, for a total length of 25 amino acids. These minigene constructs were transfected into HLA-A*11/hB2M-bearing K562 cell lines. The aforementioned procedure was performed strictly following the manufacturer’s instructions^36^.

### Induction of antigen-specific T cells by coculturing healthy donor-derived peripheral blood **lymphocytes*in vitro***

PBMCs obtained from healthy HLA-A11 donors were reviewed and approved by the Institutional Review Board (IRB) of China Medical University Hospital [Protocol number: CMUH110-REC3-014]. All procedures involving human participants were conducted in accordance with the Declaration of Helsinki and its later amendments. Written informed consent was obtained from all participants. Human peripheral blood monocyte-derived DCs were generated as described previously^35,37^. Antigen-specific T cells were generated as described previously with minor modifications^38^. After 7 days of coculture with peptide-pulsed autologous DCs, the lymphocytes were restimulated with peptide-pulsed autologous DCs in medium containing 10 ng/mL IL-7 and subsequently supplemented with 50 IU/mL rhIL-2 (Thermo Fisher). Lymphocytes were restimulated at 7-day intervals in the same manner. Half of the medium was replaced every 3 days with fresh medium containing rhIL-2 (50 IU/mL), after which the cells were expanded for 7 days. The number of cytokine-producing T cells was examined using enzyme-linked immunosorbent spot (ELISPOT) assays, and the cells were tested for cytotoxicity using AnnexinV/PI assays.

### In vitro T-cell cytotoxicity assay

The cytotoxic activity of antigen-specific cytotoxic T cells can be assessed using the AnnexinV/PI assay^35^. The formula for determining the percentage of specific lysis was as follows: cell cytotoxicity = (1 − (Ae − Ab)/(Ac − Ab)) × 100%, where Ae is the absorbance of the experimental group, Ac is the absorbance of the control group, and Ab is the absorbance of a blank well. K562-A11-HERV antigens (HLA-A11, HERV-derived peptides P1, P2, and P3) were used as peptide-specific targets.

## Data Availability

Raw RNA-seq data presented in case study 1 and 2 can be found in the NCBI database under the BioProject accession number PRJNA1171785. Proteomics data are available in the PRIDE database (https://www.ebi.ac.uk/pride/) under the accession number PXD057687.

## Code availability

All of the tools used in this study are publicly available. Comprehensive instructions of HERVOminer, including input and output descriptions and examples, are publicly available on the web interface (https://hervominer.cgm.ntu.edu.tw) and GitHub (https://github.com/jade-f-t/HERVOminer).
